# Identification of Factors Contributing to Variability in a Blood-Based Gene Expression Test

**DOI:** 10.1371/journal.pone.0040068

**Published:** 2012-07-03

**Authors:** Michael R. Elashoff, Rachel Nuttall, Philip Beineke, Michael H. Doctolero, Mark Dickson, Andrea M. Johnson, Susan E. Daniels, Steven Rosenberg, James A. Wingrove

**Affiliations:** CardioDx, Inc., Palo Alto, California, United States of America; University Medical Center Utrecht, The Netherlands

## Abstract

**Background:**

Corus CAD is a clinically validated test based on age, sex, and expression levels of 23 genes in whole blood that provides a score (1–40 points) proportional to the likelihood of obstructive coronary disease. Clinical laboratory process variability was examined using whole blood controls across a 24 month period: Intra-batch variability was assessed using sample replicates; inter-batch variability examined as a function of laboratory personnel, equipment, and reagent lots.

**Methods/Results:**

To assess intra-batch variability, five batches of 132 whole blood controls were processed; inter-batch variability was estimated using 895 whole blood control samples. ANOVA was used to examine inter-batch variability at 4 process steps: RNA extraction, cDNA synthesis, cDNA addition to assay plates, and qRT-PCR. Operator, machine, and reagent lots were assessed as variables for all stages if possible, for a total of 11 variables. Intra- and inter-batch variations were estimated to be 0.092 and 0.059 Cp units respectively (SD); total laboratory variation was estimated to be 0.11 Cp units (SD). In a regression model including all 11 laboratory variables, assay plate lot and cDNA kit lot contributed the most to variability (p = 0.045; 0.009 respectively). Overall, reagent lots for RNA extraction, cDNA synthesis, and qRT-PCR contributed the most to inter-batch variance (52.3%), followed by operators and machines (18.9% and 9.2% respectively), leaving 19.6% of the variance unexplained.

**Conclusion:**

Intra-batch variability inherent to the PCR process contributed the most to the overall variability in the study while reagent lot showed the largest contribution to inter-batch variability.

## Introduction

Peripheral blood gene expression profiling has been used to identify signatures which reflect a variety of pathological conditions, responses to pharmacological agents, and external environmental effects [1], [2], [3], [4]. Examples include gene expression tests for auto-immune and inflammatory disorders such as lupus erythematosus, heart transplant rejection, and coronary artery disease (CAD) [5], [6], [7], [8]. It has also been demonstrated that gene expression measurements can be affected by a number of *ex-vivo* events that start at the time of blood collection and continue, if care is not taken, during downstream processing [9], [10], [11]. Although an increasing number of peripheral blood gene expression signatures have been described, very few have yet been rigorously validated and implemented into broad clinical practice.

We recently described the development and clinical validation of a peripheral blood gene expression test for the assessment of the likelihood of coronary artery disease in non-diabetic patients [8], [12]. The test is a quantitative *in vitro* diagnostic performed in a Clinical Laboratory Improvement Amendments (CLIA)-certified clinical laboratory that uses the gene expression profile of circulating peripheral blood cells to estimate the likelihood of significant coronary artery disease, defined as ≥50% stenosis in a major coronary artery by quantitative coronary angiography [8].

The test score is a function of the expression levels of 23 genes which are grouped into highly correlated terms reflecting biological processes or cell types [8]. The terms, some of which are sex-specific, are weighted and combined into an algorithm which also contains the patient’s age and sex [8]. The resulting output is a gene expression score (GES) which is related to the probability of obstructive CAD. For ease of clinical interpretation, the GES is linearly transformed into a reported score ranging from 1 to 40, with increasing values representing increased probability of obstructive CAD.

Herein we describe the variability present in the clinical laboratory across a 24 month period. We assess both intra-batch and inter-batch variability, and the contributions of laboratory personnel, machine, and reagent lot to the overall variability.

## Results

**Figure 1:**
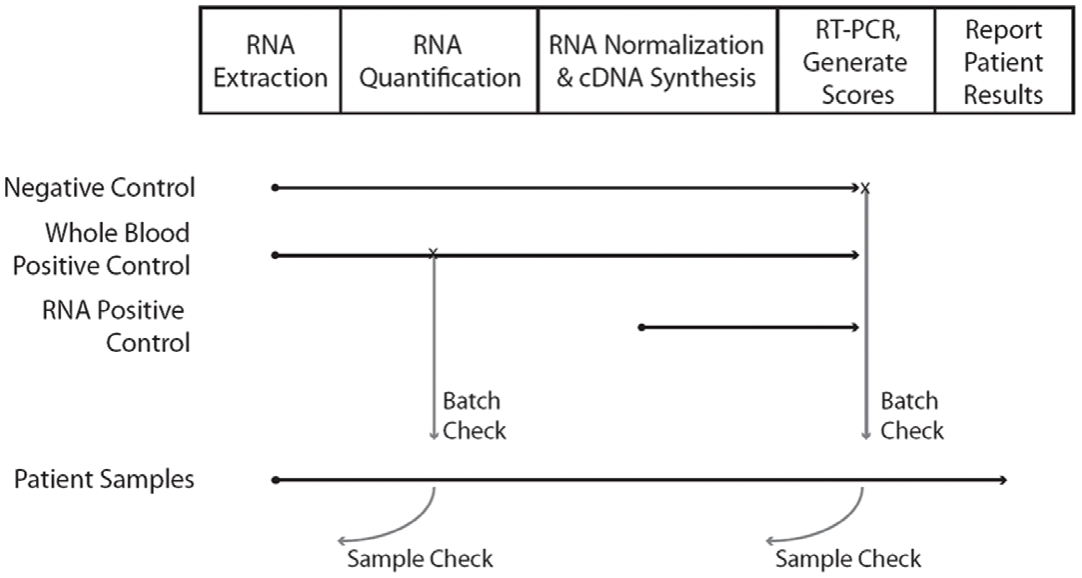
Depiction of sample flow and quality control points in the commercial laboratory. Process from whole blood sample to GES calculation consists of 4 laboratory steps and then quality control algorithm score calculation by a LIMS. Both sample controls (positive and negative) and process QC checks are used as indicated.

The clinical laboratory process flow is depicted in Figure 1. The process contains a number of QC checkpoints and controls, including the whole blood control which is run with each batch of clinical samples (Fig. 1).

### Total Variability

Total process variability was estimated using 895 whole blood control samples from the study period. The SD derived from this set of samples was 0.11 Cp units, or slightly less than 1 point on the reported GES scale (0.97 points on the 1–40 reported GES scale, 1.7% change in probability of obstructive disease, Table 1).

**Table 1:** Measurements of Total, Intra-batch, and Clinical Variability.

Type of Variability	Cp SD^1^	GES SD^1^	% Probability Change^2^
**Total Variability**	0.11	0.97	1.7%
**Intra-Batch Variability**	0.092	0.81	1.42%
**Clinical Variability**	1.19	10.5	18.4%

1 SD given in Cp units and gene expression score points (GES).

2 Percent change in probability of subject having obstructive CAD.

### Intra-Batch Variability

**Figure 2:**
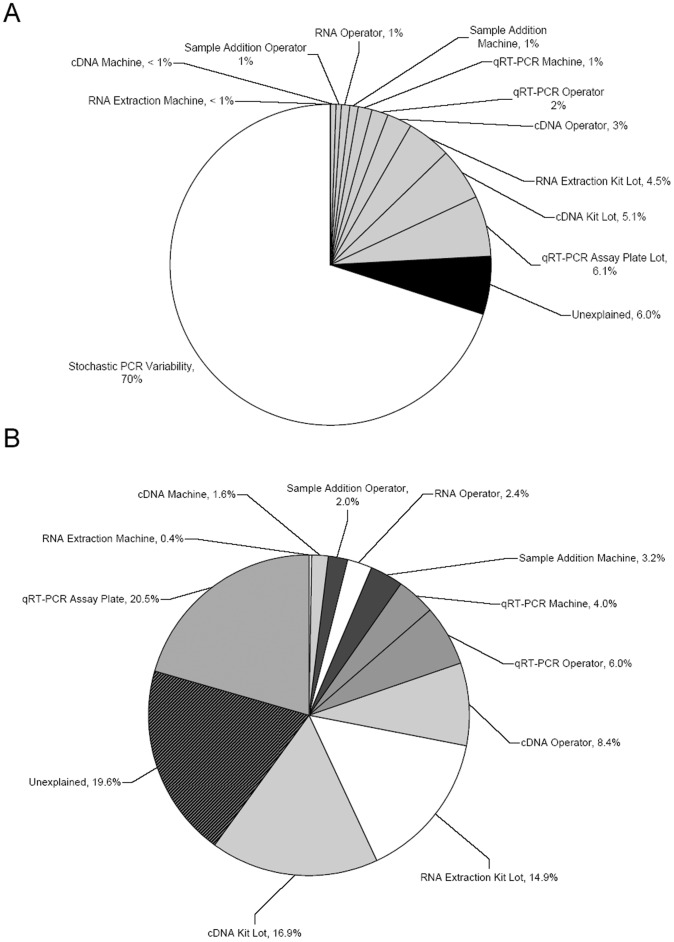
Percentages of Overall and Laboratory Variability by Component. (A) Overall variability in the laboratory process; PCR process  = 70% (speckled white); reagent lots, operators, or machines  = 24% total (grey); unaccounted = 6%. (B) Contributions of laboratory processes to non-PCR associated variability; RNA extraction (white), cDNA (light grey), qRT-PCR (mid grey), sample addition (dark grey), unexplained (black lines).

**Figure 3:**
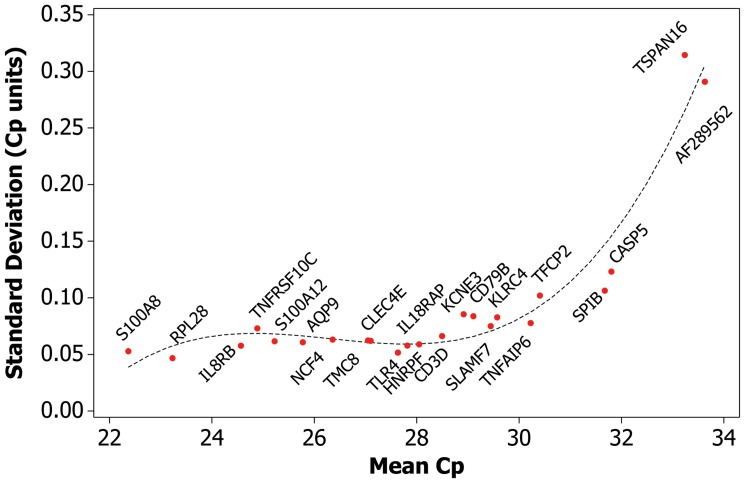
Variability, as measured by SD, increases as gene expression levels decrease, reflecting the stochastic nature of PCR. Y axis depicts SD, in Cp units; X axis depicts gene expression, in Cp units. Higher Cp units equal lower gene expression. The dashed line represents a cubic regression model fitted to the data.

In order to assess the variability inherent to the laboratory process, e.g. variability that was independent of operator, machine, and reagent lot, data was used from five batches, where the samples consisted solely of identical whole blood controls (combined N = 132). In processing these batches, single operators, machines, and reagent lots were used. The SD derived from this set of samples was 0.092 Cp units, representing ∼70% of the overall variance (Table 1, Fig. 2A). Reproducibility of expression values of the 23 gene components of the GES were examined to determine gene-specific variances. Gene-specific SD was anti-correlated with levels of gene expression, increasing with decreased expression levels (Table 2, Fig. 3). When the individual gene-specific variances were weighted, using the component weight from the GES algorithm, AF289562 contributed the most to GES variance followed by CASP5 (∼23%, ∼10% respectively, Table 2). The sum of the weighted variances equaled that of the GES (0.092 Cp units), demonstrating that variability inherent to the PCR process was the major contributor to overall variability.

**Table 2:** Gene Expression Score Component Variability.

Gene/Component	Mean Cp	SD	% Variability	AlgorithmWeight
TSPAN16^1^	33.23783329	0.314473	<1%	0
TMC8	27.0491705	0.062304	1.39%	0.069
CD3D	28.49725376	0.066184	1.47%	0.069
SPIB	31.67462259	0.106285	1.56%	0.04521
RPL28	23.23034338	0.046649	2.33%	0.154
HNRNPF	28.04762546	0.058873	2.35%	0.123
S100A8	22.36803951	0.052756	2.41%	0.14124
CD79B	29.10833979	0.083717	2.49%	0.09179
AQP9	25.77568108	0.06066	2.69%	0.137
NCF4	26.35784643	0.063076	2.80%	0.137
S100A12	25.22470751	0.061538	2.82%	0.14124
CLEC4E	27.10114833	0.061894	2.83%	0.14124
TLR4	27.6331357	0.051561	3.16%	0.189
CXCR2	24.56723698	0.057652	3.53%	0.189
TFCP2	30.40651938	0.101976	4.06%	0.123
TNFRSF10C	24.88700233	0.07295	4.47%	0.189
IL18RAP	27.81802168	0.057736	4.67%	0.24948
SLAMF7	29.44608773	0.074932	4.93%	0.203
KCNE3	28.91777707	0.085435	5.23%	0.189
KLRC4	29.5726501	0.082712	5.44%	0.203
TNFAIP6	30.22744093	0.07769	6.28%	0.24948
CASP5	31.80418539	0.123132	9.95%	0.24948
AF289562	33.62904299	0.290804	23.17%	0.246

1 Despite high having a high SD, TSPAN16 contributes little to variability in the GES due to its low weight in the control sample used; in clinical samples TSPAN16 is weighted according to sex [8].

### Variability within Individual Laboratory Processes

Variability in the clinical laboratory was assessed at the four major steps in the process: RNA extraction, cDNA synthesis, cDNA addition to assay plate, and qRT-PCR, with laboratory operator, machine, and reagent lot examined where possible (Table 3).

**Table 3:** Variability within Individual Laboratory Processes.

Method and Variable	n	p value^1^	% Variance
**RNA Extraction**			
RNA Extraction Machine	4	0.843	0.10%
RNA Extraction Kit Lot	26	<2×10^−16^	18%
RNA Operator	6	0.517	0.40%
**cDNA Synthesis**			
cDNA Synthesis Machine	4	0.074	1.6%
cDNA Kit Lot	6	**8.5**×**10^−11^**	6.9%
cDNA Operator	5	**0.012**	0.8%
**Sample Addition to Assay Plate**			
Sample Addition Machine	5	0.149	0.8%
Sample Addition Operator	8	0.358	0.9%
**qRT-PCR**			
qRT-PCR Machine	6	**0.032**	1.3%
qRT-PCR Assay Plate Lot	24	**<2**×**10^−16^**	21.8%
qRT-PCR Operator	6	**0.016**	1.5%

1 p values in bold are <0.05.

#### RNA extraction

During the study, six operators used four liquid handling robots interchangeably to extract RNA from whole blood; a total of twenty-five unique lots of RNA extraction kits were used during this period. Of the three variables, only RNA extraction kit lot contributed significantly to variability (p<0.001, Table 3).

#### cDNA synthesis

Five operators used four liquid handling robots interchangeably to perform reverse transcription on the isolated RNA; a total of six unique cDNA kit lots were used during this period. Of the three factors, cDNA kit lot showed the strongest variability, followed by cDNA operator (p<0.001; p = 0.012 respectively, Table 3). Multiple liquid handling robots introduced border-line significant variability into the process (p = 0.074, Table 3).

#### cDNA addition to assay plates

Sample addition was performed by eight operators using five liquid handling robots; neither operators nor machines introduced significant variability (Table 3).

#### qRT-PCR

Twenty four lots of assay plates were used during the study (see Methods S1 for a description of plate lot manufacturing and Table S1 for plate manufacturing quality control metrics); qRT-PCR was performed by six operators using six different thermal cyclers. All three factors demonstrated significant variability, with plate lot contributing the most followed by operator and then machine (p<0.001; p = 0.016; p = 0.032 respectively, Table 3).

### Inter-Batch Variability: Contributions of Operator, Machine, and Reagent Lot

To identify factors contributing to inter-batch variability, we examined the eleven factors listed in Table 4; these were included in a model to determine the amount of variance due to operators, machines, or reagent lots across the entire process. The most significant variable in this model continued to be cDNA kit lot, which contributed to 16.9% of the inter-batch variance (p = 0.009, Table 4, Fig. 2B). Overall, reagent lots (RNA extraction kits, cDNA kits, and assay plates) contributed the most to inter-batch variance followed by operators and then machines (52.3%,18.9% and 9.2% respectively, Fig. 2B), leaving 19.6% of the variance unexplained. When considered in the context of the overall variation, these laboratory-associated factors contributed to 24% of the overall variance, whereas 6% of the overall variance was unexplained (Fig. 2A).

**Table 4:** Components of Inter-Batch Variability Observed in the Study.

Variable	p value^1^
RNA Extraction Machine	0.935
RNA Extraction Kit Lot	0.327
RNA Operator	0.480
cDNA Synthesis Machine	0.435
cDNA Kit Lot	**0.009**
cDNA Operator	0.057
Sample Addition Machine	0.214
Sample Addition Operator	0.866
qRT-PCR Machine	0.218
qRT-PCR Assay Plate Lot	**0.045**
qRT-PCR Operator	0.061

1 p values in bold are <0.05.

### Biological versus Technical Variability

**Figure 4:**
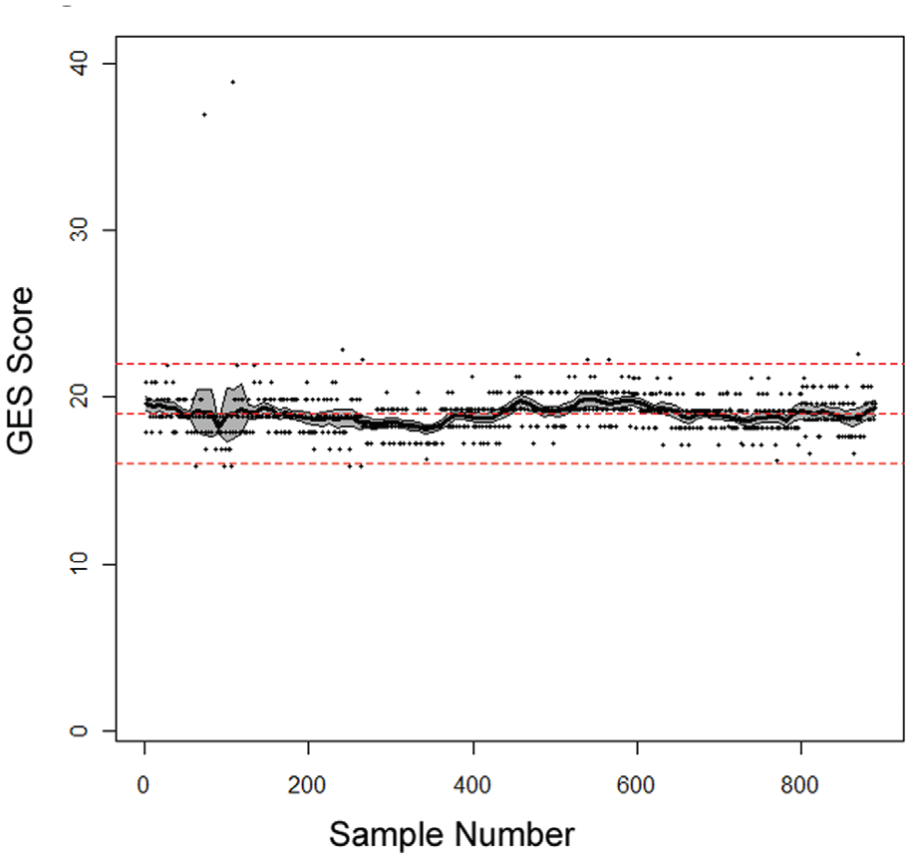
Mean Deviation of GES from Target Score Across the Course of the Study. Solid black line  =  running mean deviation of GES across the 895 samples (x axis, chronological order samples were run; y axis, GES). Middle dashed line  =  target GES; upper and lower dashed lines  =  QC boundaries ±3 points target GES. 95% CI  =  grey area.

The standard deviation (SD) across the 895 control samples was 0.11 Cp units (Table 1). In contrast, the SD across the 21,200 clinical samples run during the same period was 1.19 Cp units (10.5 points reported GES, 18.4% change in disease probability, Table 1), reflecting the biological variation in the tested population. As a quality control (QC) metric, guard-bands at ±3 points of control target reported GES were established, equating to a 6.8% change in the probability of having obstructive coronary artery disease. Of the 895 samples, 1.2% (11) fell outside this control range, resulting in the subsequent repeat of the batch of clinical samples associated with the failed control sample (Fig. 4).

## Discussion

Multi-analyte laboratory developed tests (LDTs) are often complex in nature, containing multiple sample processing steps, operators, machines and types of reagents. As such, it is important to understand both sources and amounts of process variability, as high variability can lead to erroneous test results and potentially incorrect clinical actions. Corus CAD is a multi-gene test based on the expression levels of 23 genes in whole blood and provides an estimate of the likelihood of obstructive coronary disease in non-diabetic patients. The 23 genes were selected from a larger set of genes, identified through microarray and qRT-PCR-based studies using multiple, independent cohorts [12], [13]. The genes are grouped into highly correlated meta-genes in order to minimize the impact of single gene variability [14], [15]. The meta-genes represent biological processes (e.g. apoptosis, cell death, innate and active immune responses) that are known to be associated with CAD, as well as different cell types also associated with CAD (e.g. lymphocytes, neutrophils, monocytes) [12]. The test is run in a centralized, clinical laboratory and consists of multiple processing steps, by a number of operators, and with multiple reagents. Over a two year period, control samples were used to analyze intra- and inter-batch variability in the laboratory process.

Intra-batch test variability was estimated using data from five batches consisting of identical whole blood control samples (N = 132); yielding a standard deviation of 0.092 Cp units representing approximately 70% of the overall variance (Fig 2A., Table 1). Analysis of gene-specific variability allowed us to investigate individual contributions of each gene assay. Two major factors determined how much a given gene component contributed to GES variability. The relationship between gene expression level and variability observed in PCR results is driven largely by the stochastic nature of PCR; this interaction is well-established and demonstrated in this data set, with lower-expressed genes showing higher variability (Table 2, Fig. 3) [16]. The second factor is the weight assigned to a given gene in the GES, which was derived from the gene’s performance as a classifier in algorithm development (Table 4) [12]. When selecting genes as components of a GES, caution must be taken to balance these two factors. Interestingly, the sum of the individual component variances equaled that of the total score (Table 1), suggesting intra-batch variance was driven almost exclusively by the stochastic variability inherent to the PCR process.

Inter-batch test variability (contributions from laboratory personnel, machines, and reagent lots) accounted for 24% of the overall variance (Fig. 2A). Of the three variables, reagent lots accounted for the greatest source of variability, followed by operators and machines (Fig. 2B); others have also shown that variability can be introduced into the qRT-PCR process by differing reagent lots and operators [17]. A number of steps can be instituted to diminish reagent-driven variability, including careful assessment and qualification of incoming reagent lots. Variability introduced by operators can be alleviated by operator training and well-defined SOPs, as well as through the use of liquid handling robots, whereas machine variability can be controlled through regular maintenance and calibration of the robots.

This study has limitations; it was a retrospective analysis and not prospectively designed. Approximately 6% of the overall variance remained undefined, variance that may have been accounted for in a prospectively designed study that allowed better assessment of process interactions. Despite this limitation, this “natural history” study of process variability proved worthwhile, demonstrating the majority of variability in this complex LDT is inherent in the analytical technique being used (PCR) and the importance of carefully monitoring and controlling variability from reagent lots.

The overall variability of the laboratory process is similar to what has been reported by other qRT-PCR-based molecular diagnostic tests, and is an order of magnitude less than the biological variability observed in the 21,200 clinical samples run in the lab over the same period (Table 1) [18]. Of the 895 whole blood control samples run during the study, only 11 fell outside quality control guard-bands requiring re-processing of the associated batch (Fig. 4). The overall variability of 0.11 Cp units equates to 0.97 points on the 1–40 GES scale, corresponding to a 1.7% change in the likelihood of obstructive coronary artery, an amount unlikely to alter clinical decision making (Table 1). These results demonstrate that with careful design and monitoring, complex gene-expression based LDTs such as this one can be successfully implemented in a clinical laboratory setting.

## Materials and Methods

### Ethics Statement

The whole blood used for the positive control in the study was collected from donors who had granted written consent. Ethics approval for this study was obtained from the Western Institutional Review Board (Protocol #20090362). Assessment of the gene expression scores from commercial samples was done in an anonymous fashion; the scores were analyzed without access to patient identifying information. No individual patient scores are reported, only the overall variance in the population.

### Whole Blood Collection

Whole blood samples were collected in PAXgene® RNA Blood Tubes according to the manufacturer’s instructions (PreAnalytix). Commercial samples were shipped to the clinical laboratory at 4–8°C in temperature-controlled NanoCool containers (Nanopore).

### RNA Preparation and Quantification

RNA was isolated by means of a magnetic bead based approach using the Agencourt RNAdvance Blood kit (Beckman Coulter Genomics) and the Hamilton STAR automated liquid handler (Hamilton). Extraction was performed in 96-well plates containing 400 µl of whole blood per well. Genomic DNA removal was performed using the Ambion DNase I kit (Ambion) at 37°C for 25 min. Purified RNA was quantified by absorbance at 260 nm using the NanoDrop 8000 (Thermoscientific). After RNA extraction, the concentration and purity of the RNA were assessed by measuring A_260_/_A280_ ratio; to pass samples needed an RNA concentration between 15 and 50 ng/ul and a A_260_/_A280_ ratio between 1.5 and 2.5. Samples were re-extracted if they initially failed either metric. Upon a second failure for any single metric, samples were considered a no test and not reported.

### Reverse Transcription

RNA was reverse-transcribed to cDNA using the High Capacity Reverse Transcription Kit (Life Technologies); normalization of RNA mass and reverse transcription set-up were performed on the Hamilton STAR liquid handling system. cDNA samples were diluted to a RNA equivalent of 1 ng/ul for downstream processing.

### qRT-PCR Assays

The qRT-PCR assays used in the test incorporate DNA primers and Minor Grove Binding (MGB)-containing TaqMan probes (Life Technologies); assay design is described elsewhere [8], [13].

### qRT-PCR Assay 384 Well Plate Manufacturing

Each of the 23 genes in the test was assayed in triplicate for each sample. qRT-PCR reactions were performed in 10 µL volumes consisting of 8µL of PCR reagents and 2µL of cDNA sample (1 ng/µl). PCR reagents included Universal Master Mix (Life Technologies) TaqMan assay reagents, and DEPC-treated water; after sample addition the final reaction contained 900 nM each of unlabeled primers and 250 nM of probe. Plates were manufactured using liquid handling robots (Beckman Coulter). Plates were stored at −20°C until the time of sample addition. Plates were qualified if they met pre-defined qualification metrics (Methods S1, Table S1).

### qRT-PCR

All qRT-PCR reactions were run using the Light Cycler 480 II (Roche). Cycling conditions for each plate included a 50°C incubation for 2 minutes followed by a 95°C incubation for 10 minutes. Each plate was processed through 45 cycles of 95°C for 15 seconds with a ramp time of 2°C/s and 60°C for 1 minute with a ramp time of 1°C/s. Fluorescence excitation was at 465 nm and fluorescence emission was monitored at 510 nm after each cycle for probe detection. Individual Cp values were calculated using software provided with LC480 II (Roche).

### Internal PAX Pool Blood Control

Approximately 2.5 mL of blood was collected in PAXgene® RNA Blood Vacutainer Tubes from consented donors (Western IRB Protocol #20090362). The blood/PAXgene reagent mixture from donor tubes were pooled together. Large volumes (>3 liters) were pooled per lot, requiring proper mixing to ensure solution homogeneity. After pooling and mixing, each pool was distributed into approximately 2000 1.5 ml aliquots, incubated at room temperature for a minimum of 120 min, and stored frozen at −80°C. To ascertain pool homogeneity, fifteen aliquots per lot (spanning the sequence of aliquots prepared) were assayed and the whole blood control score target was computed; batch control samples needed to be with +/−3 points of this score in order for the batch of associated clinical samples to pass quality control. Pools were qualified if they met the pre-defined qualification metrics (Methods S1).

### Statistical Methods

All analysis was performed using R, version 2.13. Standard methods were used to estimate means, SD’s, and correlations. Gene expression pre-processing and score calculations were performed as previously described (8). Analysis of variance (ANOVA) models were used to estimate the proportion of variation attributable to each factor.

## Supporting Information

Table S1 **RT-qPCR Plate Stability Metrics.**
(DOC)

Methods S1 (DOC)
