# Supplementary material for: Identification of Factors Contributing to Variability in a Blood-Based Gene Expression Test
Source: PLoS One. 2012 Jul 3;7(7):e40068. doi: 10.1371/journal.pone.0040068 (PMC3388994; doi:10.1371/journal.pone.0040068)
Supplement: Table S1 — RT-qPCR Plate Stability Metrics. (DOC) [file pone.0040068.s001.doc]

Table S1. RT-qPCR Plate Stability Metrics

| *Test* | *Passing Metric* | *Plate Stability Study Values* |
| --- | --- | --- |
| Plate to Plate Variability | SD = 0.105 to pass | SD = 0.059 |
| Well to Well Variability | SD < 0.12 to pass | SD range 0.059-0.096 |
| Quadrant Variability | All < 0.08 and > -0.08 to pass | -0.0375,0.0100, 0.0100, 0.0150 |
| Missing Wells | < 4 wells to pass | No missing wells |
