# Supplementary material for: Identification of Factors Contributing to Variability in a Blood-Based Gene Expression Test
Source: PLoS One. 2012 Jul 3;7(7):e40068. doi: 10.1371/journal.pone.0040068 (PMC3388994; doi:10.1371/journal.pone.0040068)
Supplement: Methods S1 — (DOC) [file pone.0040068.s002.doc]

**Supplemental Methods**

***Functional Testing Procedures for Internally Manufactured Materials***

*RT-qPCR Assay Plate Manufacturing*

Each 384-well plate was broken into four quadrants of 96 wells, each quadrant accommodating one commercial sample for a total of four samples per plate. Plates were manufactured in an automated process using liquid handling robots. Plates were manufactured in lots; an assay plate lot contained the same lots of reagent and was manufactured, tested, and released together for commercial use. Each lot contained multiple batches of plates (28 plates/batch), representing plates assembled during the same automated robotic run. Prior to release of a lot for commercial use, a minimum of one plate per batch was used to assess batch and lot quality.

*RT-qPCR Assay Plate Quality Control*

From the lot to be qualified, 16 assay plates are selected, generating a total of 64 scores, using control RNA as the sample source. The 16 plates must meet the following criteria in order for the lot to pass:

- To limit plate-to-plate variability, the median absolute deviation of the median plate Cps must be less than 0.105.
- For all wells in a quadrant, the difference between the location median Cp and the assay median Cp is calculated. The absolute value of the median of this difference must be less than 0.08 Cp.
- Fit a Lowess curve with the explanatory variable being the median Cps of all genes with Cps within the range of 24 to 28, and the response variable being the median absolute deviation of the Cps. The slope of the fitted model coefficient must be < 0.12.
- Within-plate, among well variability is controlled by first comparing each plate against all other plates for each individual well. For each well location on a plate, the difference between its Cp and the corresponding location median Cp is calculated (384 values in total). The median absolute deviation of this value must be less than 0.12 Cp.

*Whole Blood Quality Control*

Whole blood was derived from an internally collected pool of whole blood. To ensure internal pool homogeneity, fifteen aliquots per lot were assayed; release for commercial use required a SD of < 0.19 Cp. Two lots of the whole blood control were used, all passed the above metric (Blood Control Lot 1 SD = 0.12 RAS, Lot 2 SD = 0.06 RAS.
